# Beta-blockers for the primary prevention of anthracycline-induced cardiotoxicity: a meta-analysis of randomized controlled trials

**DOI:** 10.1186/s40360-019-0298-6

**Published:** 2019-04-25

**Authors:** Yingxu Ma, Fan Bai, Fen Qin, Jiayi Li, Na Liu, Dongping Li, Tengfang Li, Hui Xie, Da Liu, Shenghua Zhou, Qiming Liu

**Affiliations:** 1 Department of Cardiology, The Second Xiangya Hospital, Central South University, Changsha, 410011 Hunan China; 2 Department of Cardiovascular Surgery, The Second Xiangya Hospital, Central South University, Changsha, 410011 Hunan China; 3 Urological Organ Transplantation Department, The Second Xiangya Hospital, Central South University, Changsha, 410011 Hunan China; 4 Department of Cardiothoracic Surgery, The Second Xiangya Hospital, Central South University, Changsha, 410011 Hunan China

**Keywords:** Anthracycline, Cardiotoxicity, β blockers, Primary prevention

## Abstract

**Background:**

The effects of β blockers on the primary prevention of anthracycline-induced cardiotoxicity were controversial.

**Methods:**

We searched PubMed, Embase and Cochrane Library for randomized controlled trials of the comparison of β blockers versus placebo in patients undergoing anthracycline chemotherapy. This meta-analysis was performed by using random-effect models.

**Results:**

Nine hundred forty participants from 11 trials were included in this meta-analysis. β blockers led to a significant reduction in symptomatic heart failure (risk ratio [RR] 0.29, 95% CI 0.10 to 0.85). Compared with placebo, β blockers were associated with improved left ventricular ejection fraction (mean difference [MD] 4.46, 95% CI 1.77 to 7.15) and s’ (MD 0.78, 95% CI 0.01 to 1.55) in parallel with reduced left ventricular diameter (left ventricular end systolic diameter, MD -3.19, 95% CI -6.17 to − 0.21; left ventricular end diastolic diameter, MD -2.28, 95% CI 4.50 to − 0.05). β blockers also improved strain and strain rate when compared with placebo. There were no significant differences in diastolic function variables between β blockers and placebo except e’ (MD 2.33, 95% CI 0.16 to 4.51). In addition, β blockers compared with placebo reduced the risk of cardiac troponin I elevation > 0.04 ng/ml (RR 0.60, 95% CI 0.42 to 0.85). There was no marked difference in adverse events (RR 0.94, 95% CI 0.56 to 1.59) between β blockers and placebo.

**Conclusions:**

In cancer patients with anthracycline therapy, prophylactic β blockers were associated with reduced risk of heart failure, decreased left ventricular diameter, improved left ventricular systolic function, and alleviative cardiomyocyte injury.

**Electronic supplementary material:**

The online version of this article (10.1186/s40360-019-0298-6) contains supplementary material, which is available to authorized users.

## Background

Due to appropriate cytotoxic chemotherapy regimens and targeted therapy, the long-term survival rates of patients with breast cancer, leukemia, or lymphoma have been significantly improved in the past few years [1]. As the cornerstone of chemotherapy regimens, anthracycline is widely used in these patients. However, anthracycline displays cardiotoxicity that ultimately results in heart failure (HF). The mechanisms of anthracycline-induced cardiotoxicity have been unraveled, including the generation of reactive oxygen species, mitochondrial dysfunction, and activation of matrix metalloproteinase [1].

When anthracycline-induced cardiotoxicity was diagnosed, oncologists had to choose the second-line chemotherapy regimens which were associated with lower cancer-related survival rates. Furthermore, the efficacy of guideline-based treatment was poor in these patients. Only 11% of patients had full recovery, albeit with medical treatment that was recommended in the guidelines [2]. In addition, most of the patients with cardiotoxicity could not be diagnosed and treated in time because they usually had no symptoms, which was associated with poor prognosis [3]. Therefore, the primary prevention of anthracycline-induced cardiotoxicity is of paramount importance.

The impact of β blockers on cardiotoxicity prevention was under debate. A cohort study [4] enrolling 318 breast cancer patients with anthracycline and trastuzumab indicated that β blockers were associated with lower risk of new HF events (HR 0.2, 95% confidence interval [CI] 0.1 to 0.5), which suggested that prophylactic β blockers might play a role in the prevention of anthracycline-mediated cardiotoxicity. Nevertheless, a randomized controlled trial (RCT) [5] with 36-month follow-up demonstrated that there was no difference in HF between β blockers and placebo. Moreover, the results of RCTs which evaluated the effects of β blockers on cardiac function were controversial. Two studies [6, 7] indicated that β blockers inhibited the development of left ventricular (LV) systolic and diastolic dysfunction in patients with anthracycline. But OVERCOME trial [8] showed that β blockers had no effect on diastolic function, whereas CECCY trial [9] demonstrated that β blockers was associated with improved diastolic function. Against this background, this present meta-analysis was done to assess the effects of prophylactic β blockers on clinical events, cardiac anatomy, LV systolic and diastolic function, circulating biomarkers, and adverse events in patients with anthracycline chemotherapy.

## Methods

This meta-analysis was performed according to recommendations of Cochrane Handbook for Systematic Reviews of Interventions (version 5.2) [10] and Preferred Reporting Items for Systematic Reviews and Meta-Analyses (PRISMA) [11].

### Search strategy

Two authors independently searched PubMed, Embase and Cochrane Library. Cochrane Highly Sensitive Search Strategy [10] was used in the search process. Key words were “beta blocker” and “anthracycline” (details of search strategy included in Additional file 1).

### Selection criteria and quality assessment

Two reviewers respectively read titles, abstracts or full texts to identify potential articles which met inclusion criteria. Studies included in this meta-analysis were required to have: (1) RCT design; (2) cancer patients undergoing anthracycline therapy; (3) participants who were randomly assigned to β blocker or placebo groups. Trials enrolling children were excluded.

Cochrane collaboration’s tool for assessing risk of bias was used to assess the quality of included studies by two independent researchers [10]. The items included in this tool were random sequence generation, allocation concealment, blinding of participants and personnel, blinding of outcome assessment, incomplete outcome data, and selective reporting.

### Data extraction and outcome measures

Two investigators independently extracted data from included studies. Data extracted from studies included study characteristics, patient characteristics, details regarding β blocker and placebo groups, and outcome measures. The primary endpoints were clinical events (all-cause mortality and symptomatic HF). And the secondary endpoints incorporated cardiac anatomy (left ventricular end systolic diameter [LVESD], left ventricular end diastolic diameter [LVEDD], and left atrial [LA] diameter), LV systolic function (left ventricular ejection fraction [LVEF], mitral annulus tissue Doppler peak systolic velocity [s’], peak systolic strain [SS], and systolic strain rate [SSR]), LV diastolic function (peak early diastolic velocity [E], peak late diastolic velocity [A], E/A ratio, E-wave deceleration time [DT], isovolumic relaxation time [IVRT], early peak diastolic velocity of the mitral annulus [e’], E/e’ ratio, and the ratio of pulmonary vein flow peak systolic (S) and diastolic (D) velocities [S/D]), cardiac biomarkers (cardiac troponin I [cTnI] and B-type natriuretic peptide [BNP]), and adverse events.

### Statistical analysis

This meta-analysis was performed by Review Manager 5.0 and Stata 12.0. Outcome data were extracted as risk ratios (RRs) and 95% CIs or mean differences (MDs) and 95% CIs. Random-effect models were used for all outcomes because of differences in study participants and length of follow-up [10]. The Cochran’s Q test and I^2^ test were performed to assess the heterogeneity of the summary effects. If the *P* value of Cochran’s Q test was < 0.10 and I^2^ was > 50%, heterogeneity was considered to exist. [10] Publication bias was assessed by funnel plot and Begg’s test, respectively. Two substudies were done for LVEF and E/A. The first substudy quantitatively synthesized results of RCTs enrolling patients with non-selective β blockers. And the second one included RCTs in which selective β blockers were used.

Sensitivity analyses were performed for LVEF and E/A ratio to further detect clinical heterogeneity. (1) One trial [8] evaluated LVEF by means of two methods with different results. Result obtained from cardiovascular magnetic resonance (CMR) was used in a sensitivity analysis. (2) Some patients in four trials [5, 7, 8, 12] underwent radiotherapy which might cause impairment of cardiomyocytes and a part of patients in one trial [12] accepted trastuzumab which was known to be cardiotoxic. These four trials [5, 7, 8, 12] were excluded in the sensitivity analysis. (3) Patients in three trials [8, 12, 13] accepted angiotensin-converting enzyme inhibitors (ACEI) or angiotensin receptor blocker (ARB) which had cardioprotective effects. These three trials [8, 12, 13] were excluded in the sensitivity analysis.

## Results

The result of study selection process was shown in Additional file 1: Figure S1. Among 2063 articles found in the databases, 11 RCTs [5–9, 12–17] were finally included in this meta-analysis.

### Characteristics of studies and patients

Of 940 patients enrolled in this meta-analysis, 475 patients were allocated to β blocker groups. The range of the mean age was from 38.74 years to 54.3 years. Participants in 8 trials [6, 8, 9, 13–17] received carvedilol and metoprolol was used in two studies [5, 12]. And nebivolol was used in only one trial [7]. A portion of patients (19–65.6%) in 4 studies [5, 7, 8, 12] received radiotherapy. Further characteristics of included studies and patients were summarized in Table 1.

**Table 1:** Characteristics of the included randomized controlled trials

First author, year of publication	N. patients	Inclusion criteria	Follow-up (months)	Arm	Dose	Age, yrs	Female, n (%)	Radiotherapy, n (%)
Kalay, 2006 [6]	50	Patients with malignancy and planned ANT therapy	6	Carvedilol	12.5 mg, once daily	46.8 ± 14	22 (88)	0
				Placebo	Corresponding dose	49.0 ± 9.8	21 (84)	0
Georgakopoulos, 2010 [5]	125	Lymphoma patients	31	Metoprolol	100 mg, daily target doses	51.0 ± 18.0	20 (48)	8 (19)
				Placebo	Corresponding dose	49.1 ± 19.4	19 (47)	9 (23)
Kaya, 2012	45	Breast cancer patients planned anthracycline-based chemotherapy	6	Nebivolol	5 mg, once daily	51.4 ± 9.4	27 (100)	7 (26)
				Placebo	Corresponding dose	50.5 ± 11.1	18 (100)	5 (28)
Bosch, 2013 [8]	90	Acute leukemia or patients with malignant hemopathies undergoing autologous hematopoietic stem cell transplantation (HSCT) and without LVSD	6	Carvedilol	25 mg, twice daily	49.7 ± 13.9	18 (40)	12 (27)
				Placebo	Corresponding dose	50.9 ± 13.2	21 (47)	4 (9)
Liu, 2013 [13]	40	Patients with breast cancer and chemotherapy with FEC	6 Chemotherapy Circle	Carvedilol	5 mg, twice daily	53 (39–68)^a^	20 (100)	0
				Placebo	Corresponding dose	54 (37–65)^a^	20 (100)	0
Elitok, 2014 [14]	80	Patients with breast cancer and anticipated ANT therapy	6	Carvedilol	12.5 mg, twice daily	54.3 ± 9.3	40 (100)	0
				Placebo	Corresponding dose	52.9 ± 11.2	40 (100)	0
Beheshti, 2016 [16]	70	Pathologically confirmed nonmetastatic breast cancer patients	6	Carvedilol	6.25 mg, twice daily	42.0	30 (100)	0
				Placebo	Corresponding dose	39.9	40 (100)	0
Gulati, 2016 [12]	130	Women with early stage breast cancer who after breast cancer surgery were scheduled to initiate chemotherapy with FEC	10 to 61 weeks	Candesartan + Metoprolol	32 mg + 100 mg, once daily	50.0 ± 8.9	30 (100)	18 (60.0)
				Candesartan + Placebo	32 mg + corresponding dose, once daily	51.7 ± 10.7	32 (100)	19 (59.4)
				Metoprolol + Placebo	100 mg + corresponding dose, once daily	50.5 ± 9.1	32 (100)	22 (68.8)
				Placebo + Placebo.	Corresponding dose	50.8 ± 9.2	32 (100)	23 (71.9)
Jhorawat1, 2016 [15]	54	Patients diagnosed with lymphoreticular malignancy and planned for chemotherapy (CT) with regimen containing ANT	6	Carvedilol	12.5 mg, once daily	43.89 ± 15.66	4 (14.8)	0
				Placebo	Corresponding dose	38.74 ± 18.36	9 (33.3)	0
Nabati, 2017 [17]	91	Women with newly diagnosed breast cancer treated with ANT therapy	6	Carvedilol	6.125 mg, twice daily	47.10 ± 12.17	46 (100)	0
				Placebo	Corresponding dose	47.57 ± 8.75	45 (100)	0
Avila, 2018 [9]	200	Patients with HER2-negative breast cancer tumor status and therapy that included anthracycline, cyclophosphamide	6	Carvedilol	25 mg, twice a day	50.80 ± 10.10	96 (100)	0
				Placebo	Corresponding dose	52.9 ± 9.05	96 (100)	0

^a^: values are median (range); #: values are mean

Values are mean ± standard deviation

*ANT* Anthracycline, *N/A* not available

According to Cochrane collaboration’s tool, the quality of included studies was high (Additional file 1: Figure S2). Effect sizes reported in studies were distributed symmetrically (Additional file 1: Figure S3) and there was no significant bias from small studies (Begg’s test *P* = 0.13), indicating that publication bias was low.

### Clinical events

**Fig. 1:**
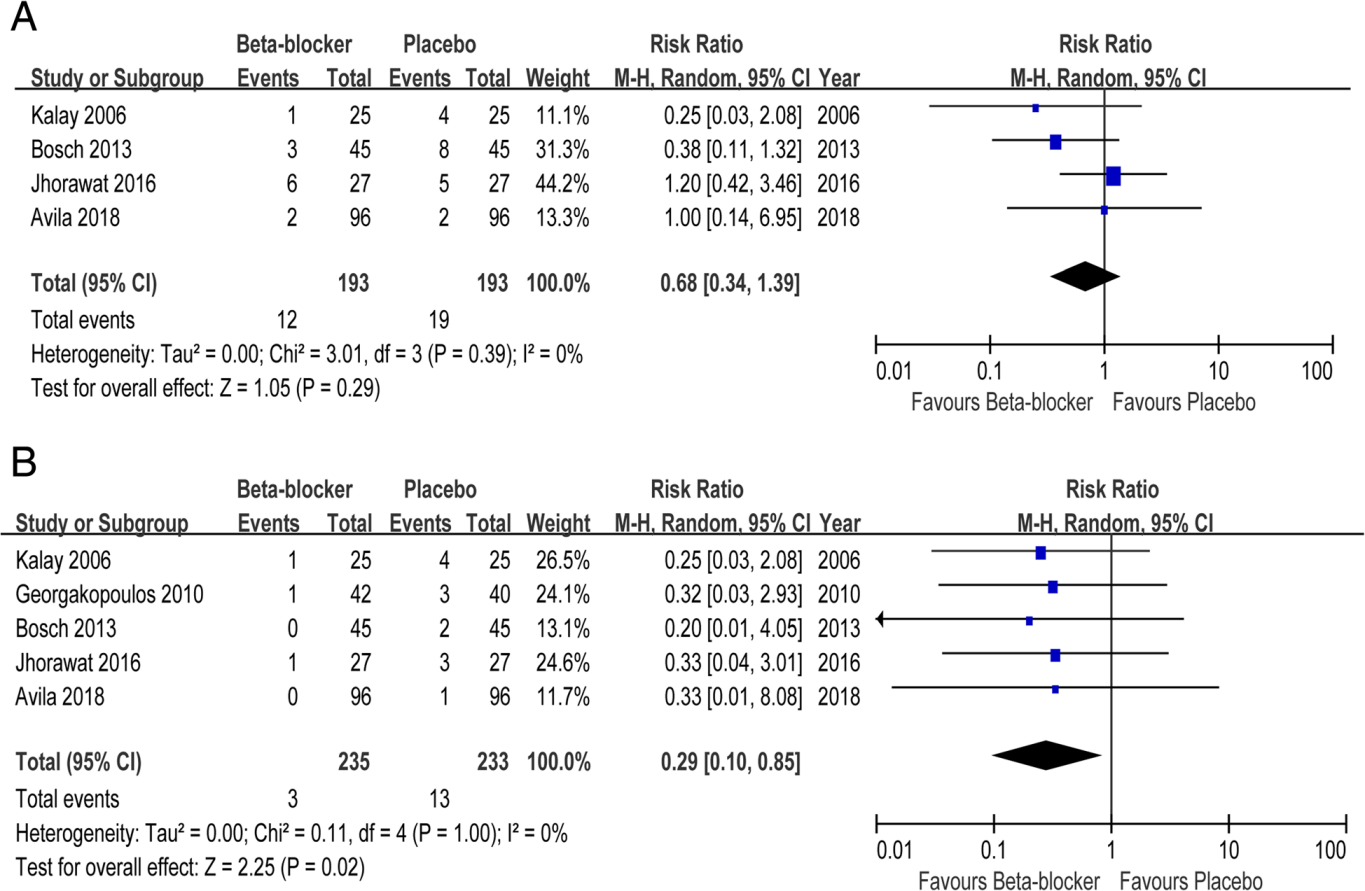
Forest plot with individual and summary estimates of the risk ratio (RR) and 95% confidence interval (CI) of clinical events. **a** All-cause mortality. **b** symptomatic HF. CI, confidence interval

There was no significant difference in all-cause mortality between β blockers and placebo (RR 0.68, 95% CI 0.34 to 1.39). Compared with placebo, β blockers reduced the risk of symptomatic HF (RR 0.29, 95% CI 0.10 to 0.85) by 71% in patients with anthracycline (Fig. 1).

### Cardiac anatomy

**Fig. 2:**
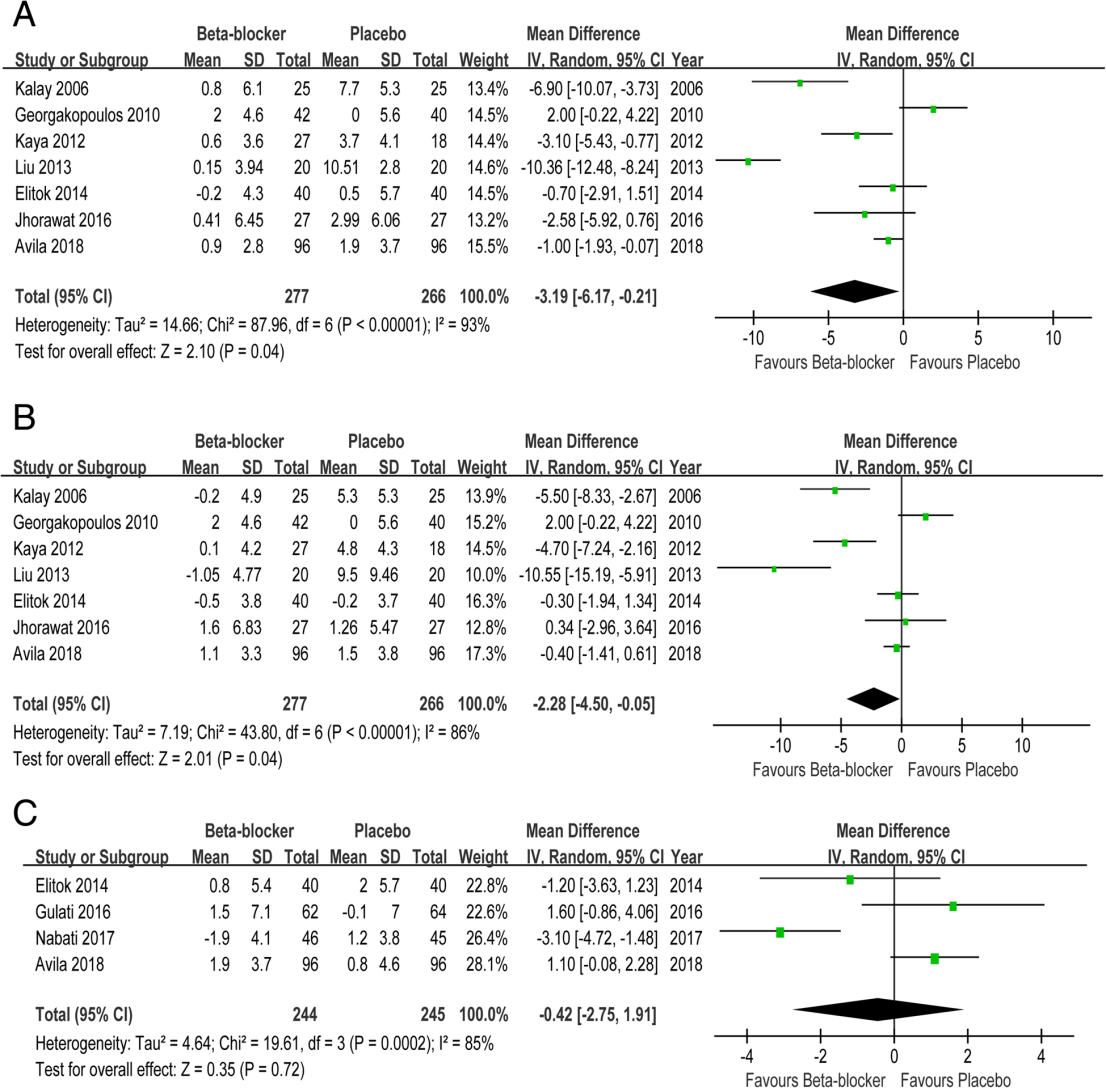
Forest plot with individual and summary estimates of the mean difference (MD) and 95% confidence interval (CI) of cardiac anatomy. **a** Change in LVESD. **b** Change in LVEDD. **c** Change in LA diameter. SD, standard deviation

β blockers compared with placebo were associated with a 3.19 mm decrease in LVESD (MD -3.19, 95% CI -6.17 to − 0.21) and a 2.88 mm decrease in LVEDD (MD -2.28, 95% CI -4.50 to − 0.05). However, no difference in LA diameter between β blockers and placebo was observed (MD -0.42, 95% CI -2.75 to 1.91) (Fig. 2).

### LV systolic functions

**Fig. 3:**
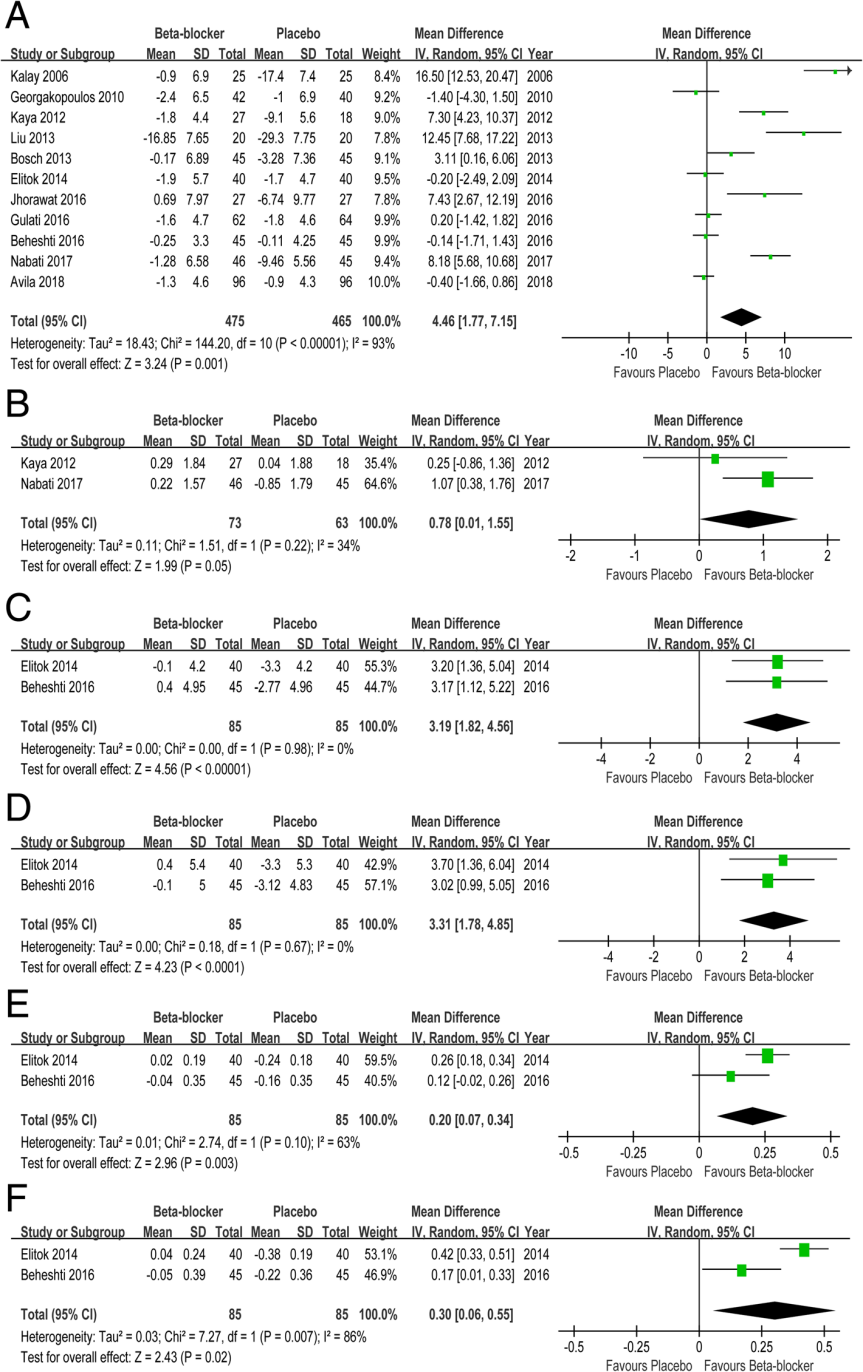
Forest plot with individual and summary estimates of the mean difference (MD) and 95% confidence interval (CI) of LV systolic functions. **a** Change in LVEF. **b** Change in s’. **c** Change in septal SS. **d** Change in lateral SS. **e** Change in septal SSR. **f** Change in lateral SSR. SD, standard deviation

There was a significant improvement in LVEF in the β blocker group versus that in the placebo group (MD 4.46, 95% CI 1.77 to 7.15). And β blockers were associated with increased s’ relative to placebo (MD 0.78, 95% CI 0.01 to 1.55). Significant protection in septal (MD 3.19, 95% CI 1.82 to 4.56) and lateral SS (MD 3.31, 95% CI 1.78 to 4.85) was observed in patients undergoing β blockers compared with placebo. And β blockers were also associated with increase septal (MD 0.20, 95% CI 0.07 to 0.34) and lateral SSR (MD 0.30, 95% CI 0.06 to 0.55) when compared with placebo (Fig. 3).

### LV diastolic functions

As shown in Additional file 1: Figure S4, e’ was significantly increased in β blocker group compared with placebo group (MD 2.33, 95% CI 0.16 to 4.51), whereas there were no significant differences in E (MD 2.37, 95% CI -6.07 to 10.81), A (MD -0.66, 95% CI -4.52 to 3.19), E/A (MD 0.04, 95% CI -0.04 to 0.11), DT (MD 5.16, 95% CI -9.04 to 19.35), IVRT (MD 0.66, 95% CI -6.32 to 7.64), E/e’ (MD -0.06, 95% CI -0.84 to 0.72), and S/D (MD 0.04, 95% CI -0.08 to 0.15) between the two groups.

### Cardiac biomarkers

**Fig. 4:**
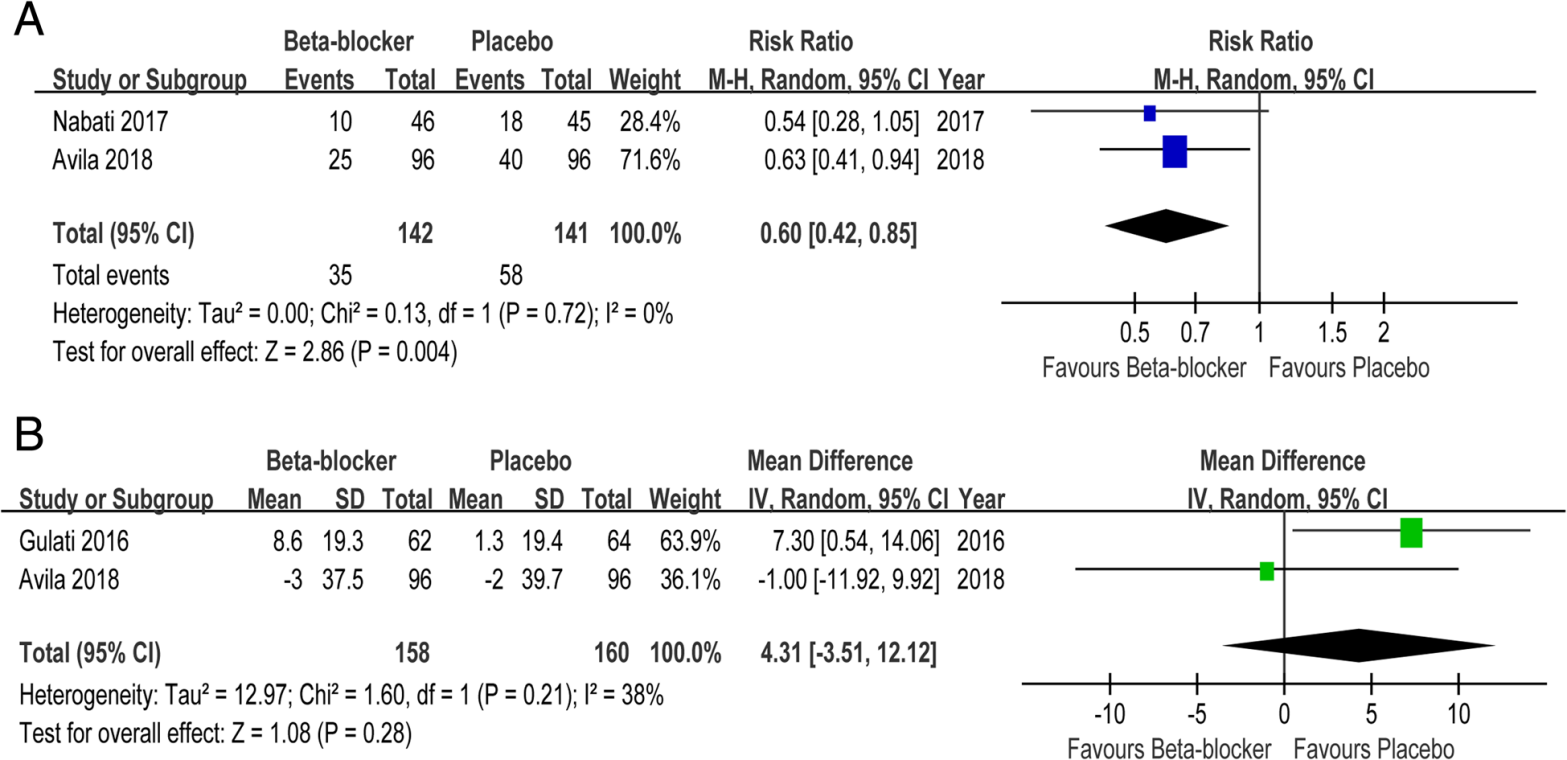
Forest plot with individual and summary estimates of the risk ratio (RR) or mean difference (MD) and 95% confidence interval (CI) of cardiac biomarkers. **a** cTnI elevation> 0.04 ng/ml. **b** Change in BNP. SD, standard deviation

Compared with placebo, β blockers reduced the risk of cTnI elevation > 0.04 ng/ml (RR 0.60, 95% CI 0.42 to 0.85) by 40%. But there was no significant difference in BNP (MD 4.31, 95% CI -3.51 to 12.12) between two groups (Fig. 4).

### Adverse events

There was no marked difference in adverse events (RR 0.94, 95% CI 0.56 to 1.59) between β blockers and placebo (Additional file 1: Figure S5).

### Substudy for patients with non-selective β blockers

The efficacy of non-selective β blockers in the prevention of anthracycline-induced cardiotoxicity was assessed in 8 trials [6, 8, 9, 13–17]. Of 687 patients included in these studies, 344 patients were assigned to carvedilol groups. When compared with placebo, carvedilol was associated with a 5.52% increase in LVEF (MD 5.52, 95% CI 1.95 to 9.10). However, there was no difference in E/A (MD -0.01, 95% CI -0.09 to 0.08) between carvedilol and placebo (Additional file 1: Figure S6).

### Substudy for patients with selective β blockers

The effects of selective β blockers on prevention of anthracycline-induced cardiotoxicity were evaluated in 3 studies [5, 7, 12] which enrolled 253 patients. Metoprolol was used in two trials [5, 12] and nebivolol was used in one trial [7]. Relative to placebo, β blockers increased LVEF (MD 1.95, 95% CI -2.57 to 6.47) with hint of significance. Yet, there was no difference in E/A between β blockers and placebo (MD 0.11, 95% CI -0.02 to 0.23) (Additional file 1: Figure S7).

### Sensitivity analyses

LVEF was measured by echocardiography and CMR in OVERCOME trial [8]. In this sensitivity analysis, the result obtained from CMR was used. Compared with placebo, β blockers were associated with a 4.50% increase in LVEF (MD 4.50, 95% CI 1.76 to 7.25).

Some patients in 4 studies [5, 7, 8, 12] received radiotherapy before randomization. And a part of patients received trastuzumab in PRADA trial [12]. Because radiotherapy and trastuzumab might result in cardiomyocyte injury, these 4 trials [5, 7, 8, 12] were excluded in this sensitivity analysis. When compared with placebo, β blockers were associated with increased LVEF (MD 5.91, 95% CI 1.88 to 9.95). Nevertheless, there was no difference in E/A (MD 0.03, 95% CI -0.04 to 0.10) between two groups.

Patients in intervention groups underwent concomitant ACEI/ARB in 3 trials [8, 12, 13]. ACEI/ARB exhibited cardioprotective effects, which might influence the evaluation of real efficacy of β blockers. On exclusion of these 3 trials [8, 12, 13], β blockers were still associated with significant improvement in LVEF (MD 4.40, 95% CI 0.98 to 7.82). But no difference was observed in E/A (MD 0.05, 95% CI -0.03 to 0.14) between β blockers and placebo.

## Discussion

To our knowledge, this is the first comprehensive meta-analysis to assess the effects of β blockers on the cardiac function in the primary prevention of anthracycline-induced cardiotoxicity. We found that β blockers were safe in the prevention of anthracycline-induced cardiotoxicity. β blockers were associated with reduced risk of symptomatic HF and improved LV systolic function in parallel with decreased LV diameter. In addition, β blockers protected the cardiomyocytes and lowered the risk of cTnI elevation.

When the cumulative dose of doxorubicin was > 700 mg/m^2^, the incidence rate of HF was 18% [3]. Hence, oncologists recommended that the maximum cumulative dose of anthracycline was 550 mg/m^2^. But Brandon et al. [18] found that low to moderate dose of anthracycline was still associated with early reduction in LVEF. Even though the cumulative dose of doxorubicin was 500 mg/m^2^, the incidence rate of HF was still as high as 16% [19]. Moreover, anthracycline-induced cardiomyopathy was associated with worse prognosis [17]. Thus, the primary prevention of cardiotoxicity was crucial to patients’ survival. Although β blockers are used in the treatment of anthracycline-induced cardiomyopathy [20], its role in primary prevention of cardiotoxicity is under debate. The aim of this meta-analysis is to assess the effects of β blockers on the prevention of cardiotoxicity.

This meta-analysis showed that β blockers did not increase the risk of adverse events, which suggested that it’s safe for patients with anthracycline to accept β blockers. And the result of CECCY trial [9] was consistent with ours. This trial indicated that there was no difference in the incidence of side effects (*P* > 0.05) between β blockers and placebo. What was more important was that concomitant β blockers could not reduce the effectiveness of anticancer drugs [14]. Besides, carvedilol could lower multidrug resistance of cancer cells [21].

This analysis demonstrated that β blockers reduced the risk of HF by 71% in patients undergoing anthracycline chemotherapy. Seicean et al. [4] showed the protective effects of β blockers on HF were more evident in breast cancer patients with anthracycline and trastuzumab. They found β blockers reduced the risk of symptomatic HF by 80% (HR 0.2, 95% CI 0.1 to 0.5). This might be because anthracycline plus trastuzumab induced more severe cardiotoxicity, which increased the susceptibility of patients to HF. Furthermore, β blockers reduced risk of HF by 50% in patients without LV dysfunction [22]. Taken together, these studies consistently showed that prophylactic β blockers were associated with a statistically significant reduction in HF risk. β blockers lowered the risk of all-cause mortality and cardiovascular death in HF patients [23]. However, it’s unclear whether the same effects of β blockers could be observed in cancer patients. This meta-analysis showed that β blockers did not reduce the incidence of all-cause mortality in patients with anthracycline. Nonetheless, the incidence of all-cause mortality was influenced by cancer-related death and the duration of follow-up was relatively short. Long-term results of included trials [5–9, 12–17] were expected.

LVEF is widely used to diagnose anthracycline-induced cardiotoxicity [2]. And it was an independent predictor of short- and long-term mortality in patients with anthracycline-induced cardiomyopathy [24]. Symptomatic or asymptomatic decline in LVEF was associated with increased mortality in patients with cardiomyopathy [25, 26]. The restoration of LVEF was accompanied by reduced risk of cardiac events [24]. Although β blockers improved LVEF in HF patients with reduced EF [23], the effects of β blockers on LVEF in cancer patients were under debate. We found that β blockers prevented deterioration of LVEF in parallel with reduction in LV volume. This was consistent with the results of a few small RCTs [6–8]. OVERCOME trial [8] demonstrated that LVEF in the intervention group remained unchanged but there was a marked reduction in LVEF in the placebo group (*P* = 0.035). However, no benefit of metoprolol on LVEF was observed in PRADA study [12]. This might be because reduction in LVEF was less than originally anticipated, leading to decreased power to detect between-group differences. Furthermore, the duration of follow-up was relatively short and the evaluation of cardiac function was terminated at the end of the cancer treatment. The benefit of metoprolol might be obvious after long-term follow-up.

Although LVEF was widely used in the clinical practice, it’s not sensitive enough in the cardiac function evaluation [16]. In the diagnosis of LV systolic dysfunction, s’ was more sensitive than LVEF, which was an independent predictor of high death risk [27]. We found that β blockers improved s’ in patients with chemotherapy. When LV systolic dysfunction occurred, the decline of strain and strain rate was earlier than that of LVEF. And strain and strain rate were more objective due to less variation among different ultrasound physicians. What was more important was that the prognostic value of LVEF in the range that was close to normal was limited. But it seemed that strain did not share this limitation [27]. Strain and strain rate were more appropriate in the assessment of LV systolic dysfunction in patients with chemotherapy because most patients in the intervention groups exhibited normal LVEF at the end of follow-up. This analysis demonstrated that β blockers improved strain and strain rate in cancer patients. These results indicated that β blockers could protect LV systolic function in patients with anthracycline.

Patients in the intervention groups accepted concomitant ACEI/ARB in 3 trials [8, 12, 13]. It’s well known that ACEI/ARB improved cardiac remodeling and protected systolic function [28]. Thus, there was the possibility that the use of ACEI/ARB caused the false positive results in this meta-analysis. In the sensitivity analysis, β blockers could significantly attenuate LVEF decline after exclusion of these 3 trials [8, 12, 13]. This result further confirmed that β blockers prevented the impairment of cardiac systolic function and the possibility of false positive results was excluded.

The effects of β blockers on diastolic function were under debate. CECCY trial [9] demonstrated that β blockers were beneficial in the primary prevention of diastolic dysfunction, but the opposite results were presented in two trials [7, 12]. Our analysis showed there were no differences in variables of diastolic function between two groups except e’. Based on these data, we could not preclude the possibility that β blockers were associated with improved diastolic function. Because the guideline recommended that the diagnosis of diastolic dysfunction should be based on E/e’, e’, LA maximum volume index, and peak velocity of tricuspid regurgitation (TR) in the presence of normal LVEF [29] and LVEF of patients in the included studies was > 50%. However, LA maximum volume index and peak TR velocity were not evaluated in the included studies. Meanwhile, positive result was obtained from e’ and negative result was obtained from E/e’. Thus, we’re able to make a specific conclusion on the basis of the mixed results. We expected that further studies which included all the variables.

Cardiac biomarkers were reliable to detect cardiomyocyte injury. Anthracycline could increase cTnI levels [30]. Elevated cTnI was associated with high risk of cardiovascular mortality and HF in patients with chemotherapy [30]. And the high cTnI level was the important predictor of cancer therapeutics related cardiac dysfunction [31]. This analysis showed that β blockers could lower the risk of cTnI elevation > 0.04 ng/ml. This indicated that prophylactic β blockers could prevent the cardiomyocyte injury. Moreover, we found that BNP levels were not affected by β blockers, which was expected. Because β blockers were associated with increased BNP levels in healthy population [32], which might lead to the elevation of BNP levels in the patients of the intervention group even though they had no HF.

All the patients who were allocated to the non-selective β blocker groups accepted carvedilol. This analysis demonstrated that the protective effect of carvedilol on LVEF was more obvious. This might be because the antioxidant properties of carvedilol were stronger than other types of β blockers [6]. In addition, the metabolites of carvedilol in the body exhibited the antioxidant properties and the antioxidant activity was 50–100 times greater than carvedilol [33]. This implied that carvedilol might be the first choice in the prevention of cardiotoxicity. Unfortunately, selective β blockers seemed not to protect the systolic function in this meta-analysis. However, physicians could not exclude the possibility that selective β blockers played a role in the prevention of systolic dysfunction because the reason why this subgroup showed negative results was that two trials [5, 12] about metoprolol showed negative results. And these two trials [5, 12] had several limitations which were discussed in the previous section. In the meantime, a post hoc analysis of PRADA trial [34] indicated that the troponin response was attenuated by metoprolol (*P* = 0.019), which suggested metoprolol alleviated the cardiomyocyte injury in patients with anthracycline. Prophylactic metoprolol might be beneficial to these patients. In addition, Kaya et al. [7] showed that nebivolol protected systolic and diastolic function. The large RCTs about metoprolol and nebivolol with long-term follow-up were expected.

### Limitations

There were a few limitations in this meta-analysis. Firstly, the sample size of this study is relatively small. Therefore, it is possible that type II error may be responsible for some of the negative findings. Secondly, we were unable to evaluate the effects of β blockers on global longitudinal strain and circumferential strain because the included studies only reported strain and strain rate of some segments. Thirdly, all the included studies did not report the cardiovascular mortality rate. This might be because the duration of follow-up was relatively short in most included studies. We expected the long-term results of these trials.

## Conclusions

When compared with placebo, prophylactic β blockers were associated with lower risk of HF, reduced LV diameter, improved LV systolic function, and decreased risk of cTnI elevation. And β blockers did not increase the risk of adverse events relative to placebo. β blockers might be beneficial in the primary prevention of anthracycline-induced cardiotoxicity.

## Additional file

Additional file 1: **Supplementary Methods**. **Figure S1.** Flow chart showing the process of study selection and numbers of studies included. **Figure S2.** Risk of reporting bias for each study included in this meta-analysis. **Figure S3.** Assessment of publication bias by the funnel plot. **Figure S4.** Forest plot with individual and summary estimates of the mean difference (MD) and 95% confidence interval (CI) of LV diastolic functions. **Figure S5.** Forest plot with individual and summary estimates of the risk ratio (RR) and 95% confidence interval (CI) of adverse events. **Figure S6.** Forest plot with individual and summary estimates of the mean difference (MD) and 95% confidence interval (CI) of LV systolic and diastolic functions in the substudy for patients with non-selective β blockers. **Figure S7.** Forest plot with individual and summary estimates of the mean difference (MD) and 95% confidence interval (CI) of LV systolic and diastolic functions in the substudy for patients with selective β blockers. (DOCX 1924 kb)

## Abbreviations

ACEI: Angiotensin-converting enzyme inhibitors
ARB: Angiotensin receptor blocker
BNP: B-type natriuretic peptide
CI: Confidence interval
CMR: Cardiovascular magnetic resonance
cTnI: Cardiac troponin I
HF: Heart failure
LA: Left atrial
LV: Left ventricular
LVEDD: Left ventricular end diastolic diameter
LVEF: Left ventricular ejection fraction
LVESD: Left ventricular end systolic diameter
MD: Mean difference
RCT: Randomized controlled trial
RR: Risk ratio
