# Supplementary material for: Beta-blockers for the primary prevention of anthracycline-induced cardiotoxicity: a meta-analysis of randomized controlled trials
Source: BMC Pharmacol Toxicol. 2019 Apr 25;20:18. doi: 10.1186/s40360-019-0298-6 (PMC6485127; doi:10.1186/s40360-019-0298-6)
Supplement: Supplementary file 1 — Supplementary Methods. Figure S1. Flow chart showing the process of study selection and numbers of studies included. Figure S2. Risk of reporting bias for each study included in this meta-analysis. Figure S3. Assessment of publication bias by the funnel plot. Figure S4. Forest plot with individual and summary estimates of the mean difference (MD) and 95% confidence interval (CI) of LV diastolic functions. Figure S5. Forest plot with individual and summary estimates of the risk ratio (RR) and 95% confidence interval (CI) of adverse events. Figure S6. Forest plot with individual and summary estimates of the mean difference (MD) and 95% confidence interval (CI) of LV systolic and diastolic functions in the substudy for patients with non-selective β blockers. Figure S7. Forest plot with individual and summary estimates of the mean difference (MD) and 95% confidence interval (CI) of LV systolic and diastolic functions in the substudy for patients with selective β blockers. (DOCX 1924 kb) [file 40360_2019_298_MOESM1_ESM.docx]

**Supplementary Data**

**Supplementary Methods**

**Search Strategy**

PubMed

#1 “beta blocker” OR “beta blockers” OR atenolol OR propranolol OR metoprolol OR arotinolol OR betaxolol OR bevantolol OR bisoprolol OR carteolol OR carvedilol OR celiprolol OR sotalol OR nebivolol OR labetalol OR esmolol

#2 anthracycline OR aclarubicin OR daunorubicin OR daunomycin OR plicamycin OR rubidomycin OR doxorubicin OR adriamycin OR pharmorubicin OR epirubicin OR idarubicin OR valrubicin OR mitoxantrone OR pirarubicin OR amrubicin OR aclacinomycin OR darubicin

#3 #1 AND #2

Embase

#1 'beta adrenergic receptor blocking agent' OR 'beta blocker' OR 'beta blockers' OR atenolol OR propranolol OR metoprolol OR arotinolol OR betaxolol OR bevantolol OR bisoprolol OR carteolol OR carvedilol OR celiprolol OR sotalol OR nebivolol OR labetalol OR esmolol

#2 'anthracycline antibiotic agent' OR anthracycline OR aclarubicin OR daunorubicin OR daunomycin OR plicamycin OR rubidomycin OR doxorubicin OR adriamycin OR pharmorubicin OR epirubicin OR idarubicin OR valrubicin OR mitoxantrone OR pirarubicin OR amrubicin OR aclacinomycin OR darubicin

#3 #1 AND #2

Cochrane Library

#1 “beta blocker” OR “beta blockers” OR atenolol OR propranolol OR metoprolol OR arotinolol OR betaxolol OR bevantolol OR bisoprolol OR carteolol OR carvedilol OR celiprolol OR sotalol OR nebivolol OR labetalol OR esmolol

#2 anthracycline OR aclarubicin OR daunorubicin OR daunomycin OR plicamycin OR rubidomycin OR doxorubicin OR adriamycin OR pharmorubicin OR epirubicin OR idarubicin OR valrubicin OR mitoxantrone OR pirarubicin OR amrubicin OR aclacinomycin OR darubicin

#3 #1 AND #2


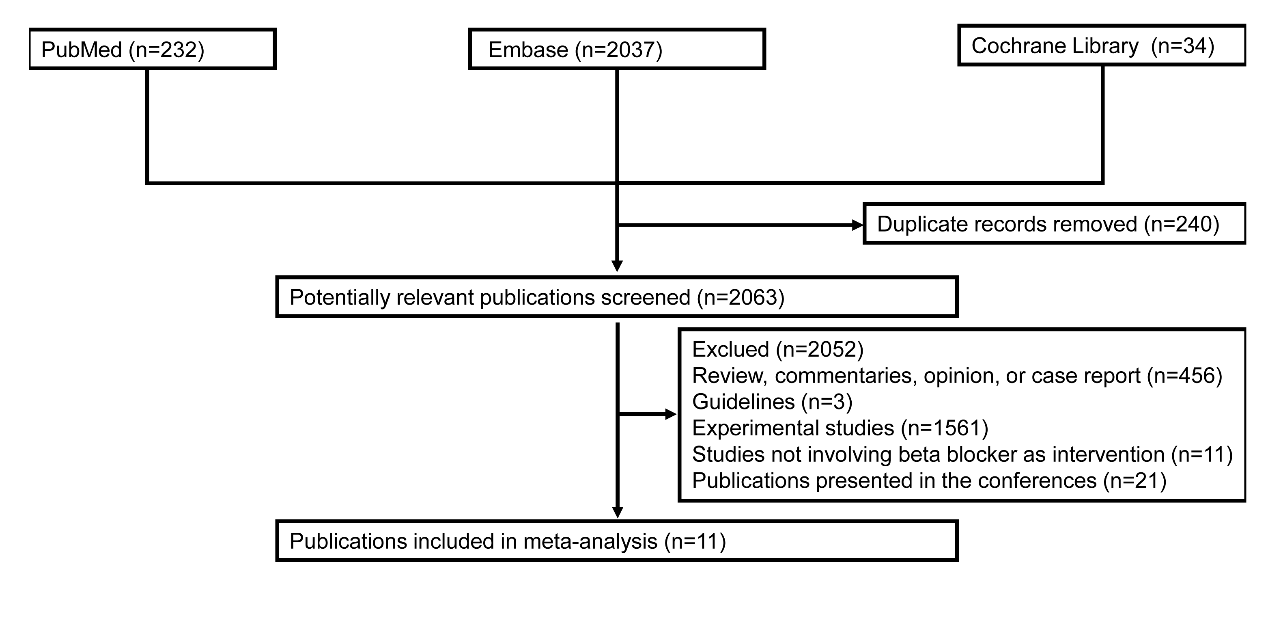


**Supplementary Figure S1** Flow chart showing the process of study selection and numbers of studies included.

**
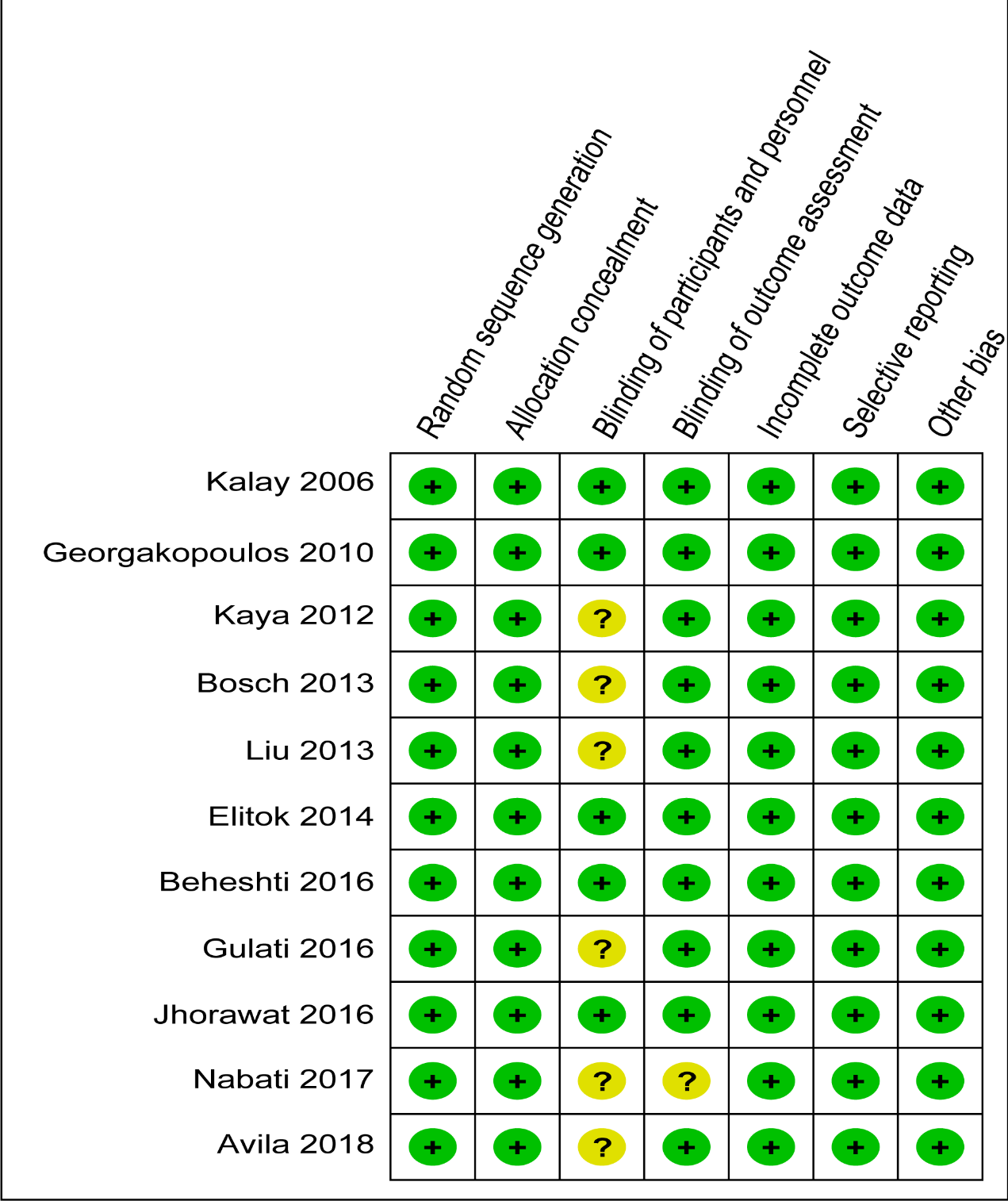
**

**Supplementary Figure S2** Risk of reporting bias for each study included in this meta-analysis.

**
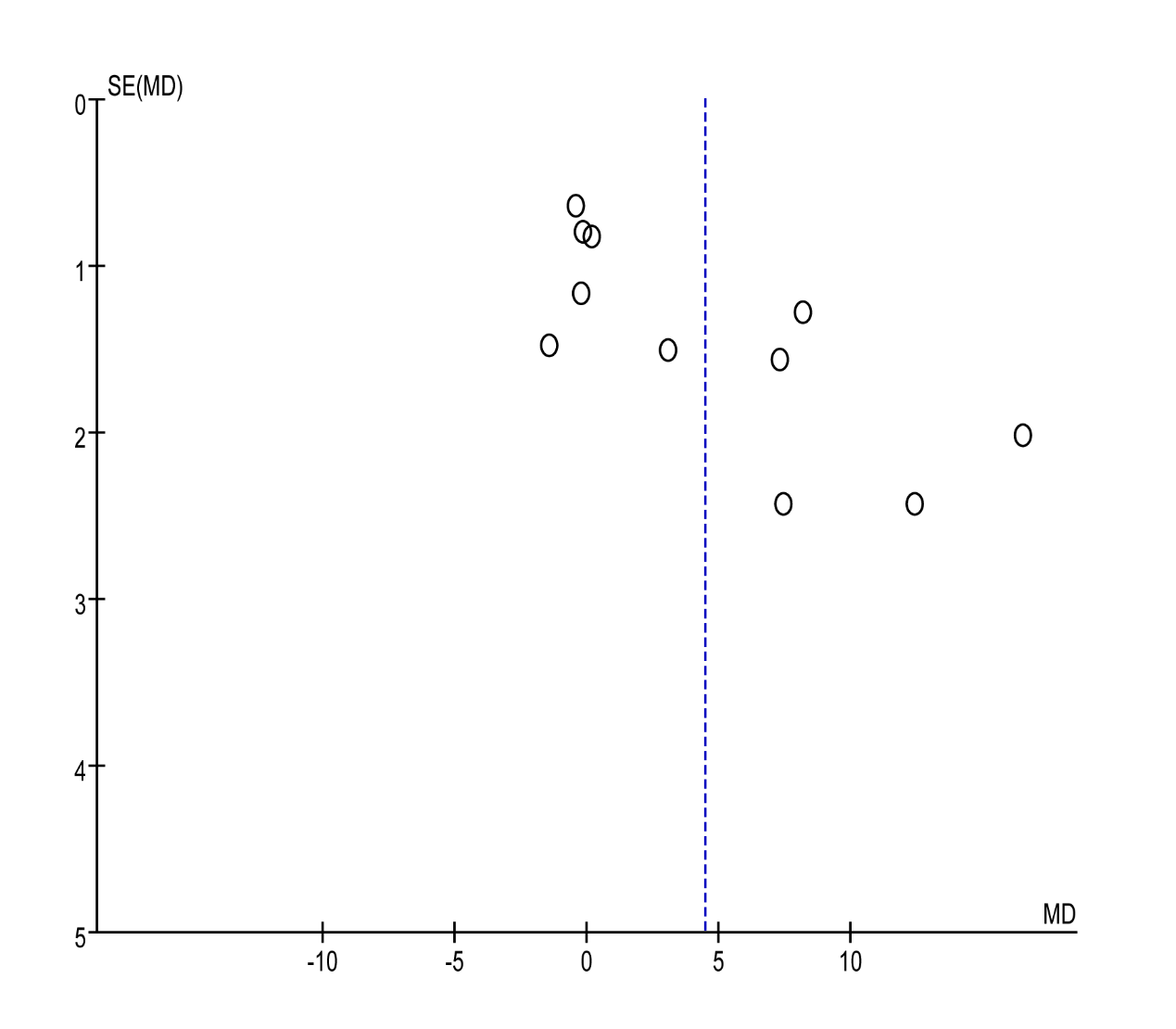
**

**Supplementary Figure S3** Assessment of publication bias by the funnel plot.

MD, mean difference; SE, standard error.

**
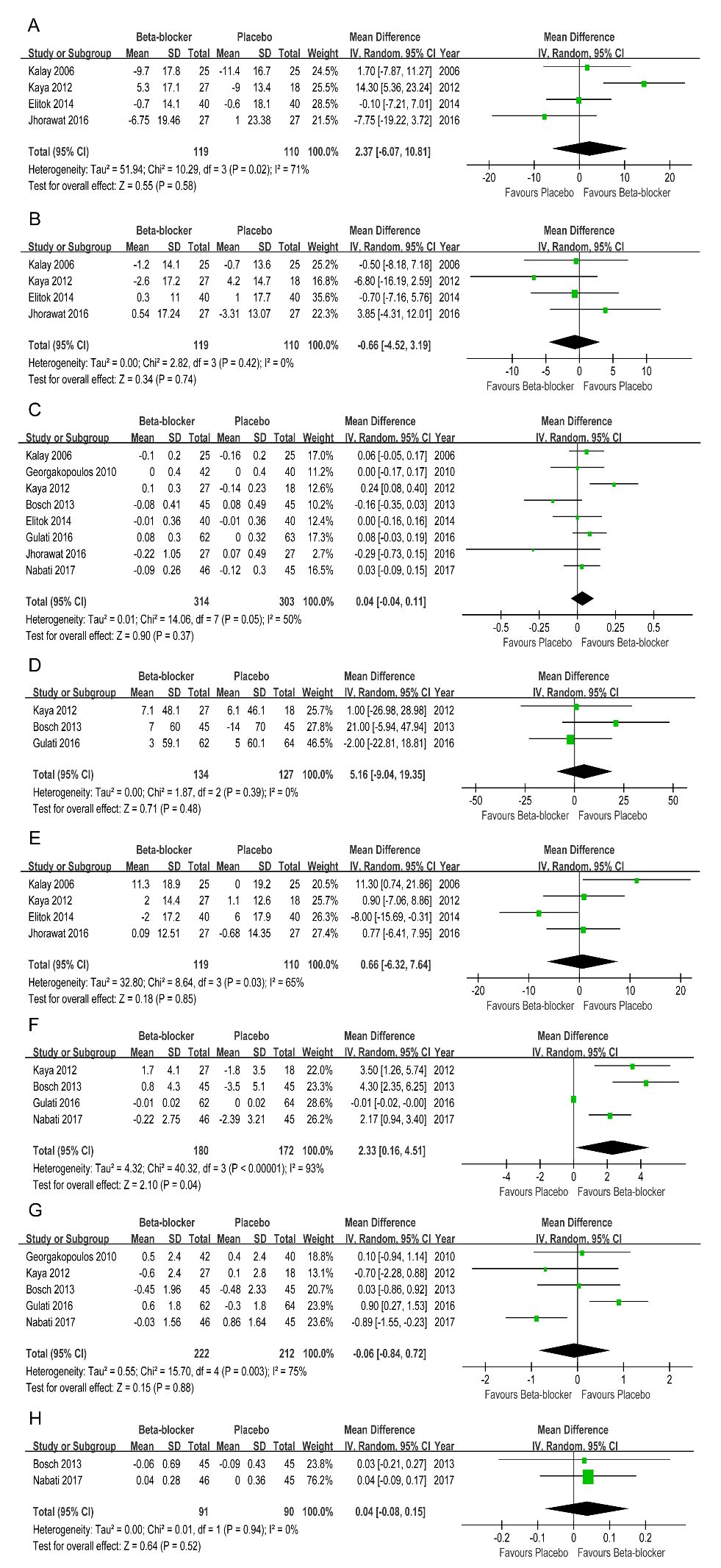
**

**Supplementary Figure S4** Forest plot with individual and summary estimates of the mean difference (MD) and 95% confidence interval (CI) of LV diastolic functions.

**(A)** Change in E. **(B)** Change in A. **(C)** Change in E/A. **(D)** Change in DT. **(E)** Change in IVRT. **(F)** Change in e’. **(G)** Change in E/e’. **(H)** Change in S/D.

SD, standard deviation.

**
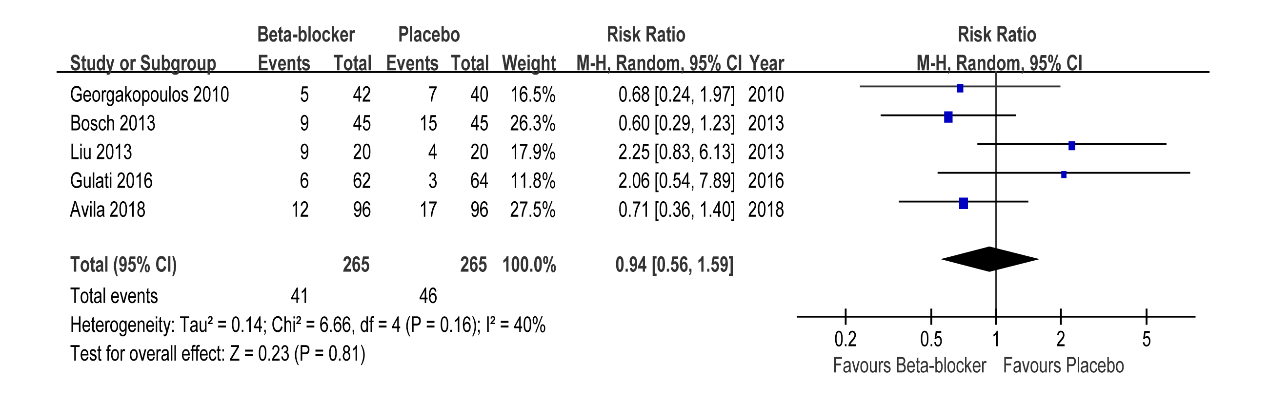
**

**Supplementary Figure S5** Forest plot with individual and summary estimates of the risk ratio (RR) and 95% confidence interval (CI) of adverse events.

CI, confidence interval.


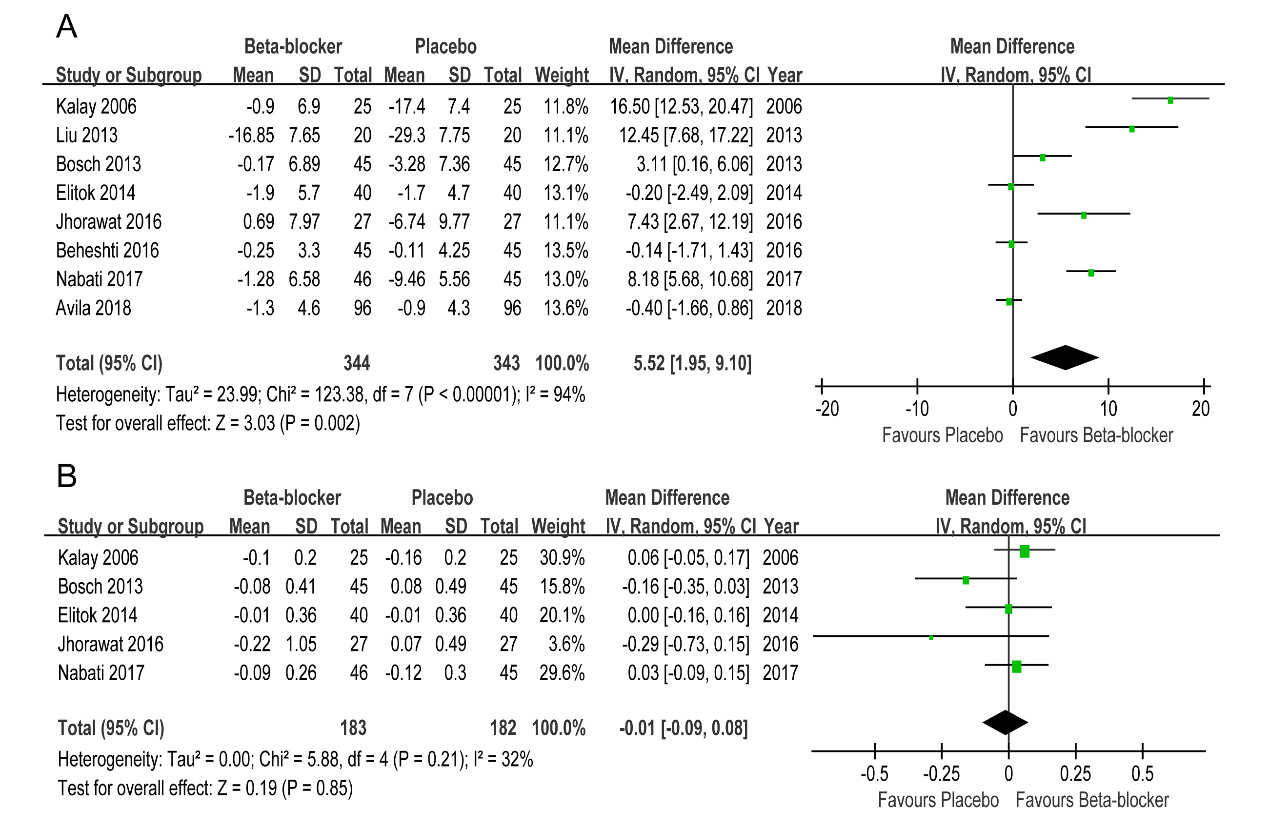


**Supplementary Figure S6** Forest plot with individual and summary estimates of the mean difference (MD) and 95% confidence interval (CI) of LV systolic and diastolic functions in the substudy for patients with non-selective β blockers.

**(A)** Change in LVEF. **(B)** Change in E/A.

SD, standard deviation.


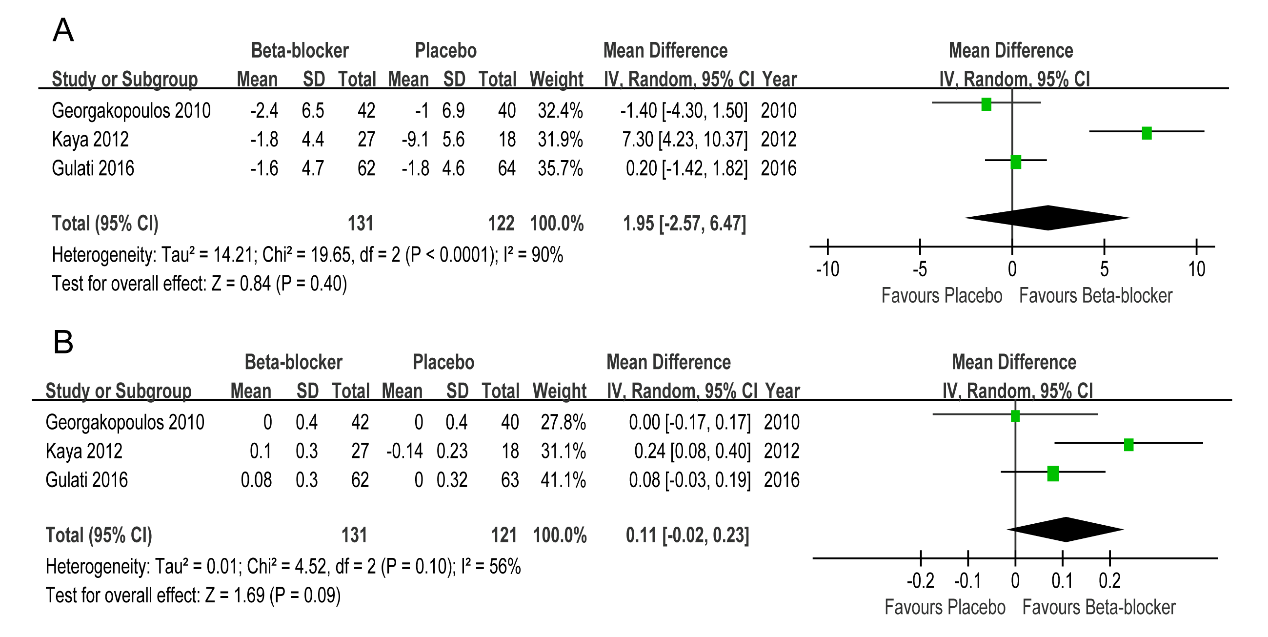


**Supplementary Figure S7** Forest plot with individual and summary estimates of the mean difference (MD) and 95% confidence interval (CI) of LV systolic and diastolic functions in the substudy for patients with selective β blockers.

**(A)** Change in LVEF. **(B)** Change in E/A.

SD, standard deviation.
